# Efficient Machine Reading Comprehension for Health Care Applications: Algorithm Development and Validation of a Context Extraction Approach

**DOI:** 10.2196/52482

**Published:** 2024-03-25

**Authors:** Duy-Anh Nguyen, Minyi Li, Gavin Lambert, Ryszard Kowalczyk, Rachael McDonald, Quoc Bao Vo

**Affiliations:** 1 School of Software and Electrical Engineering Swinburne University of Technology Hawthorn Australia; 2 School of Computing Technologies RMIT Melbourne Australia; 3 Iverson Health Innovation Research Institutes and School of Health Sciences Swinburne University of Technology Hawthorn Australia; 4 Baker Heart & Diabetes Institute Melbourne Australia; 5 STEM University of South Australia Adelaide Australia; 6 Systems Research Institute Polish Academy of Sciences Warsaw Poland

**Keywords:** question answering, machine reading comprehension, context extraction, covid19, health care

## Abstract

**Background:**

Extractive methods for machine reading comprehension (MRC) tasks have achieved comparable or better accuracy than human performance on benchmark data sets. However, such models are not as successful when adapted to complex domains such as health care. One of the main reasons is that the context that the MRC model needs to process when operating in a complex domain can be much larger compared with an average open-domain context. This causes the MRC model to make less accurate and slower predictions. A potential solution to this problem is to reduce the input context of the MRC model by extracting only the necessary parts from the original context.

**Objective:**

This study aims to develop a method for extracting useful contexts from long articles as an additional component to the question answering task, enabling the MRC model to work more efficiently and accurately.

**Methods:**

Existing approaches to context extraction in MRC are based on sentence selection strategies, in which the models are trained to find the sentences containing the answer. We found that using only the sentences containing the answer was insufficient for the MRC model to predict correctly. We conducted a series of empirical studies and observed a strong relationship between the usefulness of the context and the confidence score output of the MRC model. Our investigation showed that a precise input context can boost the prediction correctness of the MRC and greatly reduce inference time. We proposed a method to estimate the utility of each sentence in a context in answering the question and then extract a new, shorter context according to these estimations. We generated a data set to train 2 models for estimating sentence utility, based on which we selected more precise contexts that improved the MRC model’s performance.

**Results:**

We demonstrated our approach on the Question Answering Data Set for COVID-19 and Biomedical Semantic Indexing and Question Answering data sets and showed that the approach benefits the downstream MRC model. First, the method substantially reduced the inference time of the entire question answering system by 6 to 7 times. Second, our approach helped the MRC model predict the answer more correctly compared with using the original context (*F*_1_-score increased from 0.724 to 0.744 for the Question Answering Data Set for COVID-19 and from 0.651 to 0.704 for the Biomedical Semantic Indexing and Question Answering). We also found a potential problem where extractive transformer MRC models predict poorly despite being given a more precise context in some cases.

**Conclusions:**

The proposed context extraction method allows the MRC model to achieve improved prediction correctness and a significantly reduced MRC inference time. This approach works technically with any MRC model and has potential in tasks involving processing long texts.

## Introduction

Health professionals and the general public have a high demand for information and knowledge in the health care domain. However, traditional information retrieval (IR) systems such as PubMed do not provide precise responses to queries because they only return a list of relevant abstracts or full-text publications, which the user must read and interpret themselves. Thus, question answering (QA) systems that provide direct answers are preferred to enable the best use of evidence in clinical care [1]. Machine reading comprehension (MRC) is the task of predicting an answer to a question based on an input context. A large body of MRC research is based on extractive methods, where the answer is a text span from the input context that best answers the question [2]. Although extractive answers are confined to the input context, they are grounded and sensible. Current approaches to MRC tasks that achieve state-of-the-art performance on open-domain data sets such as the Stanford Question Answering Data Set [2] (SQUAD) have been shown to not work well when the input is long [3]. When the context is very long, it is difficult for the MRC models to compute attention scores for the context; as a result, answer prediction is poor. In biomedical QA applications, the context of a question is often embedded within a scientific article, which may comprise hundreds of sentences, such as in the Question Answering Data Set for COVID-19 (COVID-QA) [4] and the Biomedical Semantic Indexing and Question Answering (BioASQ) data sets [5], where the context is an entire scientific article. The length of the contexts in the COVID-QA data set is much greater than that of the contexts in the SQUAD data set (6119 vs 153 tokens or words), and the answers in COVID-QA are also longer than those in the SQUAD data set (14 vs 3 tokens). Consequently, existing MRC models typically predict less accurately and more slowly in the biomedical domain.

The work by Min et al [6] proposed a sentence selection–based method for extracting minimal context from documents in QA. Their strategy was to find the exact sentence that contained the answer. Experiments on the TriviaQA [7], NewsQA [8], and SQUAD [2] data sets showed improvement in both the inference speed and answer *F*_1_-score. However, one could argue that this strategy might not be suitable for complex domains such as health care, as the information provided in the ground truth answer sentence might not be sufficient to make accurate predictions.

Other studies have proposed techniques aimed at reducing the length of input context by identifying only the relevant sentences necessary for answering the question within the document. Context extraction has been used in QA and other natural language processing tasks such as machine translation and text summarization [6,9-11]. Instead of having the model process a long document, which can be inefficient in some domains, the context extraction task aims to focus on relevant parts of the document and to confine the main model to working solely on those parts. By shortening the input, the inference time and task accuracy can be improved.

Yang et al [12] proposed combining an IR model (Anserini) with a Bidirectional Encoder Representations from Transformers (BERT) [13] MRC model, where the IR model selected the paragraphs most similar to the question, and the selected texts were passed to the BERT model for more accurate answer predictions. Although the method was effective on the SQUAD data set, the simple IR strategy for context extraction based on textual similarity is less likely to work well on more complex domains where there is little overlap between the question and the context.

A previous study [14] proposed a sentence classification approach to predict which sentences from the document (context) constituted the best answer. The sentence selection approach was also used in the study by Min et al [6] to shorten the context before passing it as an input to the MRC model. The approaches in the studies by Kang et al [9] and Wang and Jin [10] used reinforcement learning models to learn to select a context from a long document for MRC and machine translation tasks, respectively. The approaches in the studies by Min et al [6], Wang and Jin [10], and Wang and Jin [15] proposed several techniques for selecting a short context from a long document before passing it to an MRC model. These approaches relied on a sentence selection mechanism to construct a new context. The common rationale behind the sentence selector was based on the semantic similarity between the sentence and the ground truth answer. Thus, some studies [6,9,10] have trained a model that predicted the existence of the ground truth answer in a sentence. In complex domains, information from multiple sentences may be needed to answer a question, although most of the important sentences have little semantic similarity with the answer. Similarly, although the sentence classification method proposed in the study by Min et al [6] works well for less complicated domains where one-sentence contexts are sufficient to answer questions, the method may struggle to find contexts consisting of multiple sentences in more complex domains because it does not consider the information expressed in multiple sentences. The reinforcement learning–based approach in the studies by Kang et al [9] and Wang and Jin [10] takes into account the currently selected sentences when considering another sentence; however, the models were trained to recognize the existence of the ground truth answer in the selected sentences.

In this study, we introduce a novel approach to context extraction based on sentence selection. We estimate the usefulness of each sentence within its surrounding context for answer prediction. We showed that our approach can select the correct context while keeping it much shorter than the original article.

## Methods

### Designing the Baselines

Intuitively, a shorter input context reduces the MRC inference time, but does it improve the prediction as well, assuming that the shortened context contains the relevant information and answer? We used the fine-tuned Robustly Optimized BERT Pretraining Approach (RoBERTa) model in the study by Möller et al [4] and the COVID-QA data set to test this hypothesis and develop the baselines for our proposed approach.

MRC task prediction correctness was measured using the *F*_1_-score, which is the harmonic mean of precision and recall of the prediction. Precision is obtained by dividing the number of correctly predicted tokens by the number of predicted tokens. Recall is equal to the number of correct tokens divided by the number of ground truth tokens. Another metric that may be used to evaluate an MRC task is exact match (EM), which is the percentage of the test cases that are exactly the same as the ground truth.

We selected three types of input context as baseline approaches, representing different quality levels of the context:

1. Original article: Worst-case scenario in which the MRC model takes the longest time to predict.
2. Paragraph retriever: We used the paragraph retriever to select the top-k paragraphs (k=6) and concatenate them to obtain the selected context. This strategy can be considered as a simple context extraction method. In most cases, the paragraph retriever can extract the relevant information to the question while keeping the extraction considerably shorter than the original article; details of the retrieval model are provided in the *Cosine Similarity for Paragraph Retrieval and Sentence Utility* section.
3. Target paragraph: We extracted the paragraph containing the ground truth answer sentence and used it as the input context for the MRC model. Intuitively, the target paragraph contains the relevant information as well as the answer to the question, so it can be considered the near-perfect solution. We assumed that the target paragraph is close to the best context selected by humans.

The results in Table 1 confirm the hypothesis that a more precise input context leads to better MRC performance. As the input length is reduced, inference times (s) of the paragraph retriever baseline and the target paragraph baseline are significantly lower than those of the original article baseline. In contrast, while keeping the relevant information, the more precise input context makes it easier for the MRC model to predict the answer, as can be seen from the target paragraph baseline *F*_1_-scores and EM scores. The paragraph retriever baseline did not show any clear improvement in terms of the prediction correctness.

**Table 1:** The *F*_1_-scores and inference time of the different context extraction baselines on the Question Answering Data Set for COVID-19.

	COVID-QA		
	*F*_1_-score	Exact match score	Time (s)
Original article	0.724	0.462	54.6
Paragraph retriever	0.724	0.47	8.8
Target paragraph	0.757	0.494	2.2

### Estimating Sentence Utility Based on Answer Correctness and Confidence Score

A previous study by Min et al [6] proposed to predict whether each sentence contains the answer and extracted the highly probable ones as the input context. However, we show that this logic is not suitable for more complex domains such as health care. The example in Table 2 was taken from the COVID-QA data set and tested using the fine-tuned RoBERTa MRC model. Different input contexts were fed to the RoBERTa model, and the output prediction and confidence scores were observed. In the first 3 cases, although the contexts were all relevant to the question, the MRC model could not make a prediction because insufficient information was given (*F*_1_-score≈0), especially in the third case where the input context contained the ground truth answer. Only in the later cases, where the input contains both the answer sentence and the sentences with relevant information, the MRC model could make correct predictions (high *F*_1_-score of 0.92). In the last case, although the MRC model made a highly accurate prediction, the input context clearly contained excessive redundant information. We present a method to assess the usefulness of a sentence within a given context for answering a question. The method aims to assign high utility to sentences that are either relevant to the question or contain the answer, while assigning low utility to irrelevant sentences.

**Table 2:** Example of the confidence score and prediction correctness changing with different context inputs: input contexts created by sentences relevant to the question are fed to the fine-tuned Robustly Optimized BERT Pretraining Approach (RoBERTa) model, and its predictions and confidence scores are observed; *F*_1_-score is calculated based on the predicted answer and the ground truth answer. When a useful sentence is added to the context, both the prediction correctness (*F*_1_-score) and the model’s confidence increase. The input question and ground truth answer of this example are “Why are lactic acid bacteria considered an attractive delivery system for a live influenza vaccine?” and “considered safe and exhibits an adjuvant-like effect on mucosal and systemic immunity,” respectively.

Number	Input context	Predicted answer	*F*_1_-score	Confidence score
1	Recently, LAB^a^ presenting influenza virus antigens have been studied [3,18,19].	No answer	0	2.12
2	For mucosal immunization, LAB is a more attractive delivery system than other live vaccine vectors, such as Shigella, Salmonella, and Listeria [20,21].	No answer	0	0.84
3	It is considered safe and exhibits an adjuvant-like effect on mucosal and systemic immunity [18,22,23].	No answer	0	2.75
4	Recently, LAB presenting influenza virus antigens have been studied [3,18,19]. For mucosal immunization, LAB is a more attractive delivery system than other live vaccine vectors, such as Shigella, Salmonella, and Listeria [20,21].	For mucosal immunization	0.14	3.70
5	For mucosal immunization, LAB is a more attractive delivery system than other live vaccine vectors, such as Shigella, Salmonella, and Listeria [20,21]. It is considered safe and exhibits an adjuvant-like effect on mucosal and systemic immunity [18,22,23].	It is considered safe and exhibits an adjuvant-like effect on mucosal and systemic immunity	0.92	5.53
6	Recently, LAB presenting influenza virus antigens have been studied [3,18,19]. It is considered safe and exhibits an adjuvant-like effect on mucosal and systemic immunity [18,22,23].	It is considered safe and exhibits an adjuvant-like effect on mucosal and systemic immunity	0.92	3.48
7	Recently, LAB presenting influenza virus antigens have been studied [3,18,19]. For mucosal immunization, LAB is a more attractive delivery system than other live vaccine vectors, such as Shigella, Salmonella, and Listeria [20,21]. It is considered safe and exhibits an adjuvant-like effect on mucosal and systemic immunity [18,22,23].	It is considered safe and exhibits an adjuvant-like effect on mucosal and systemic immunity	0.92	11.18
8	Recently, LAB presenting influenza virus antigens have been studied [3,18,19]. For mucosal immunization, LAB is a more attractive delivery system than other live vaccine vectors, such as Shigella, Salmonella, and Listeria [20,21]. It is considered safe and exhibits an adjuvant-like effect on mucosal and systemic immunity [18,22,23]. Anchoring of the target protein to the cell surfaces of LAB is primarily intended to use in mucosal vaccines. The transmembrane protein pgsA^b^ is one of the poly-cglutamate synthetase complexes of *Bacillus subtilis* [17,24,25], which is a well-studied anchor protein and is able to fuse the target protein to its C terminus and stabilize the complex by anchoring it in the cell membrane. As sM2^c^ is a highly conserved and promising target for a universal vaccine and CTA1^d^ is strong mucosal adjuvant, in this study, we developed constructs using a consensus sM2 gene reconstituted from the analysis of H1N1, H5N1, and H9N2 influenza viruses (no transmembrane domain) with or without the fusion of CTA1. To achieve this, we used a novel expression vector that can express a pgsA gene product as an anchoring matrix. Our target antigens, sM2 and CTA1, were displayed on the surface of *Lactobacillus casei*, and the oral or intranasal administration of recombinant *L. casei* induced systemic and mucosal immune responses that have the potential to protect against the lethal challenges of divergent influenza subtypes.	It is considered safe and exhibits an adjuvant-like effect on mucosal and systemic immunity	0.92	11.81

^a^LAB: lactic acid bacteria.

^b^pgsA: phosphatidylglycerol phosphate synthase.

^c^sM2: Hepatic Fibrosis Susceptibility Due To Schistosoma Mansoni Infection.

^d^CTA1: cholera toxin A1.

In Table 2, we show the correlation between the prediction correctness, the MRC model’s confidence score, and the input context. Intuitively, if adding a sentence to the input context leads to an improved prediction, that sentence is useful, and its usefulness is more or less proportional to the increase in prediction correctness. In Table 2, when adding the sentence in case 3 to the sentences in cases 1 and 2 as the input context, the MRC model made a big improvement in the *F*_1_-score (cases 5-7). This indicates that the sentence in case 3 was important for answering the question (because it contained the ground truth answer). In contrast, this also shows that the sentence in case 3 alone was insufficient, and some utility existed in the sentences in cases 1 and 2.

The confidence score is output by the MRC model alongside the predicted answer, which represents how strongly the model believes that the prediction is the correct answer. Intuitively, the confidence score also reflects the quality of the context; it increased more when important sentences were added to the input context and did not increase or increased slightly when redundant sentences were added (Table 2 shows the confidence score changing with different input contexts in a similar way with prediction correctness).

On the basis of these observations, we realize a strong relationship between the usefulness of a sentence and the increase in answer correctness and MRC model confidence. Thus, we propose 2 methods to calculate the utility value, the usefulness of a sentence within a context, as follows:

*u*1 = *F*1 (*q*,*D*) − *F*1 (*q*,*D*\*c*) **(1)**

*u*2 = *Conf* (*q*,*D*) − *Conf* (*q*,*D*\*c*) **(2)**

where *q* is the question, *c* is the sentence from which the utility is being calculated, *D* is the context containing *c* (usually the article or document), *Conf* is the confidence score output by the MRC model, and *F*1 is the *F*_1_-score of the predicted answer for a question-context pair.

Calculating *u*_1_ requires a known ground truth answer, and calculating *u*_2_ requires running the MRC model multiple times. Therefore, to obtain utility scores at inference time, we train a model for approximating the utility scores. From the COVID-QA data set, we randomly selected 1519 questions to generate training data for the proposed utility model, and the remaining 500 questions were used as the test set. From the training set, 500 questions were randomly selected as the validation set, which was used for the parameter optimization. Approximately 120,000 triplets of question-sentence-context were sampled, and the fine-tuned RoBERTa model [4] was used to calculate 2 types of utility values according to equations 1 and 2, which are the training signals of the model. The contexts were selected according to the following strategy to simulate different levels of quality:

- The context contained sufficient information to answer the question, and the target paragraph was chosen for this scenario.
- The context contained relevant but insufficient information to answer the question. To simulate this scenario, the context was composed of 1 to 5 sentences around the ground truth sentence, except itself.
- The context contained somewhat relevant information but was not needed to answer the question. We selected the paragraph adjacent to the target paragraph as the context for this case.
- The context was completely irrelevant to the question, which was simulated by choosing the paragraph furthest from the target paragraph or from another article as the input context.

We selected the sentences such that the utility values covered a wide range, based on the intuition that the closer the sentence is to the ground truth sentence, the higher its utility.

**Figure 1:**
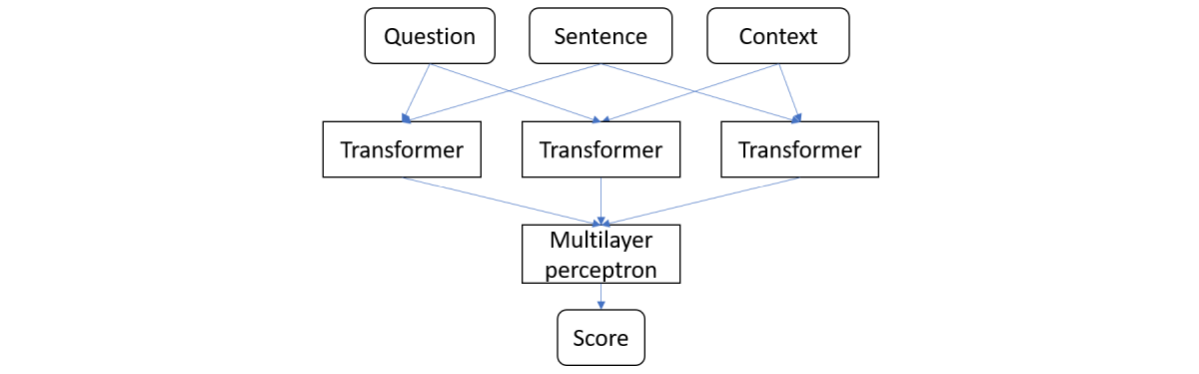
Proposed utility model structure.

The utility model architecture is shown in Figure 1, and 2 versions of the utility model were trained: F1 based and confidence based.

### Cosine Similarity for Paragraph Retrieval and Sentence Utility

**Figure 2:**
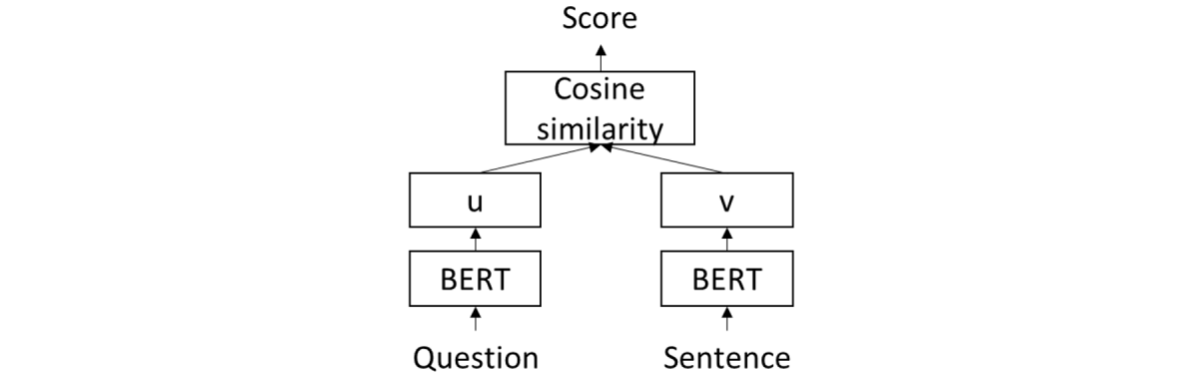
Cosine similarity model structure. BERT: Bidirectional Encoder Representations from Transformers.

The articles in the COVID-QA data set are structured into paragraphs. The information necessary for answer prediction is often contained within 1 paragraph. On the basis of this observation, we trained a simple retrieval model based on BERT [16] and cosine similarity for selecting paragraphs relevant to the question. The retrieval model is a biencoder (Figure 2) that estimates the cosine similarity between the encodings of the question and a paragraph. On the basis of the similarity scores, paragraphs were ranked, and the top-k paragraphs were selected. The cosine similarity score can also be calculated for a pair of question-sentence, which also represents the relevance of a sentence. As a result, we used the cosine similarity score as the third type of sentence utility *u*_3_.

### Context Extraction Strategy

The paragraph retriever can produce a similarity score between the question and 1 sentence from the article; thus, it can be used as the third type of utility value *u*_3_. Then, we combined the F1-based utility values, confidence-based utility values, and cosine similarity scores in an ensemble manner:

*u* = (ω_1_*u*_1_ + ω_2_*u*_2_ + ω_3_*u*_3_) / (ω_1_ + ω_2_ + ω_3_) **(3)**

where *u*_1_, *u*_2_, and *u*_3_ are utility scores produced by the F1-based utility model, confidence-based utility model, and retrieval model, respectively; ω_1_, ω_2_, and ω_3_ are the weights of the utility models, respectively.

**Figure 3:**
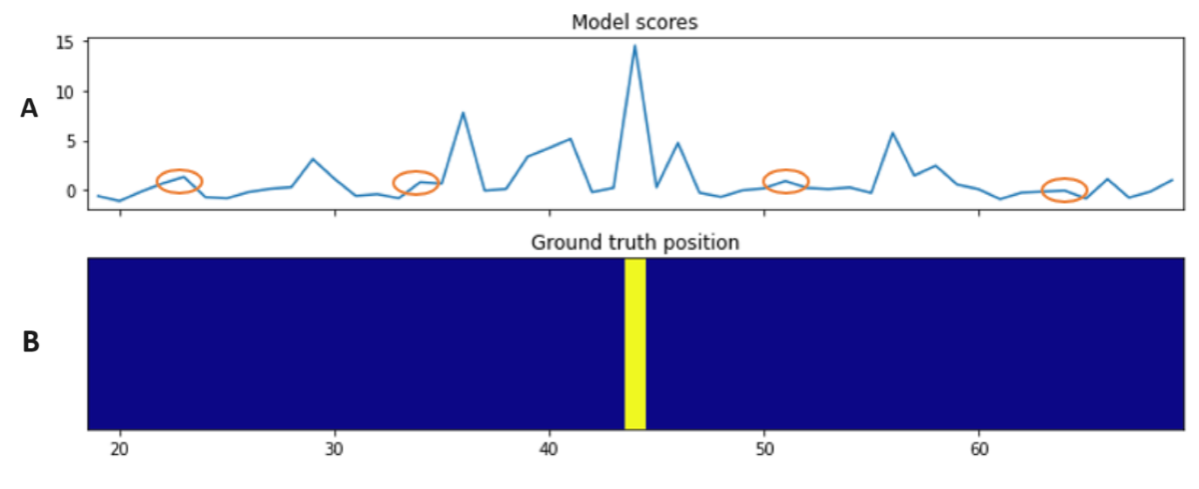
Example of sentence utility distribution in an article. Subgraph A shows the sentence utility values in an article estimated by the confidence-based utility model; subgraph B shows the location of the sentence containing the ground truth in the article.

Textbox 1 shows the algorithm for context extraction using sentence utility. The rationale was to find potential locations in the article that might contain the necessary information for answer prediction. Once trained, the utility model can estimate the usefulness of sentences to new questions and contexts. Naturally, the ground truth sentence often has the highest utility value in the article; therefore, at test time, the utility model is used to estimate the utility of the sentences to locate potential useful context positions. After estimating the utility value of each sentence in an article, one should focus on the *peaks* as they are likely close to the ground truth sentence. As not all peaks are useful (eg, the peaks marked in circles in Figure 3 are likely irrelevant to answering the question), only the highest peaks are of interest. Thus, we set parameter *h* as the threshold percentage of the selected peaks compared with the tallest one. After the peaks were identified, we selected the neighboring *w* sentences of each peak (*w* sentences before and *w* sentences after the peak sentence).

**Context extraction algorithm.**

- Input: question *q* and article *D*
- Output: context *c*
- Parameters:
  *h*∈[0,1]: threshold percentage of peaks’ values compared to the tallest
  *w*∈N*: number of sentences to be selected from the peak
  *k*∈N*: number of retrieved paragraphs
  ω1, ω2, ω3∈(0,1): utility model weights

1. Use paragraph retriever to select top-*k* paragraphs in *D*.
2. Estimate the utility and cosine similarity scores for each sentence in the retrieved paragraphs using the F1-based and confidence-based utility models, and the paragraph retriever, calculate the ensemble utility score using equation 3.
3. Select all peak sentences, whose values are ≥h*max(scores).
4. Select surrounding sentences of the peaks which are within a distance of *w* sentences.
5. Context *c* is all the selected sentences.

### Parameter Optimization

There are 6 parameters in our proposed context extraction algorithm: *k, w, h, ω_1_, ω_2_, and ω_3_*. We applied the Bayesian optimization method to the validation data set to determine the best combination of parameters. As it takes considerable time to obtain answer *F*_1_-score for the validating data set, it is impractical to use answer *F*_1_-score as an optimization objective. Instead, we constructed a loss function based on 2 subobjectives:

1. Ground truth sentence selection accuracy (*obj*_1_): The percentage of samples in which the selected context contained the ground truth sentence. As our algorithm is based on identifying sentences with peak utility scores, we considered finding the ground truth sentences as the most important factor in our algorithm.

- Context *F*_1_-score (*obj*_2_): In addition to locating ground truth sentences accurately, the algorithm needs to limit the length of the selected context to reduce inference time and potentially increase answer prediction correctness. For this, we chose to use the target paragraph (the paragraph containing the ground truth sentence) as the second subobjective to optimize the algorithm. Intuitively, the target paragraph is a near-ideal scenario, as shown in the next section, allowing the MRC model to achieve higher prediction correctness and fast inference time. We compared the selected context with the target paragraph and calculated its context *F*_1_-score as follows:

*context precision = (# correct words) / (# words in selected context)*
**(4)**

*context recall = (# correct words) / (# words in target paragraph)*
**(5)**

*context F1 = (2 × precision × recall) / (precision + recall)*
**(6)**

Thus, we constructed the loss function for the optimization method as follows:


*L = − (α × obj_1_) + (1 − α) × (obj_2_)*
**(7)**

where *α* is a hyperparameter signifying the weight of the first objective.

With each α value in range [0.05,0.1,...,0.95], a Bayesian optimization process [17] was applied to obtain a set of optimized parameters. Finally, the lowest objective value *L* was achieved with α=.95, and we chose the corresponding parameters for our context extraction algorithm.

### Ethical Considerations

This study did not involve any data nor methods that require ethical considerations.

## Results

### Data

The COVID-QA data set [4] contains 2019 question-answer pairs, 1500 (74.29%) of which were used to generate training data for the proposed utility model, and the remaining (n=519, 25.71%) were used for evaluation. We also evaluated our approach on the BioASQ data set [5]. We extracted 342 factoid-type questions from the BioASQ 7b-10b data sets to evaluate our proposed context extraction method. The MRC model used for this data set was the RoBERTa model fine-tuned on BioASQ data [18]. Table 3 presents the results.

**Table 3:** The *F*_1_-score and inference time of the different context extraction settings; the Robustly Optimized BERT Pretraining Approach model was fine-tuned separately on the Question Answering Data Set for COVID-19 (COVID-QA) and Biomedical Semantic Indexing and Question Answering (BioASQ) data set.

	COVID-QA				BioASQ		
	*F*_1_-score	Exact match score	Time (s)	*F*_1_-score		Exact match score	Time (s)
Original article	0.724	0.462	54.6	0.651		0.576	0.94
Paragraph retriever	0.724	0.47	8.8	0.7		0.608	0.14
Target paragraph	0.757	0.494	2.2	0.731		0.664	0.28
Our approach	0.744	0.498	7.9	0.704		0.626	0.16

Experiments on the COVID-QA data set were performed on a GTX1050 graphics processing unit (GPU), and experiments on the BioASQ data set were performed on an A100 GPU.

### Paragraph Retrieval Accuracy

The performance of the paragraph retriever was measured by top-*k* accuracy, which is the percentage of test cases where the paragraph containing the ground truth answer was among the *k* highest-scoring paragraphs.

Paragraph retrieval accuracy of the test set with k = 1, 2, ..., 10 are 0.904, 0.95, 0.972, 0.978, 0.986, 0.992, 0.992, 0.994, 0.994, 0.994, respectively.

### MRC Performance With Context Extraction

The context extraction model’s performance was measured on the test set using different metrics: answer *F*_1_-score, EM, and inference time. According to our experiments, the inference times of the paragraph retrieval model and the utility model were 0.02 seconds and 0.3 seconds, respectively.

We used the parameters selected from the optimization method described in the *Parameter Optimization* subsection of the *Methods* section for our context extraction algorithm, which was responsible for selecting a new shorter context from the original article and then passing it to the RoBERTa model [4].

The results in Table 3 show the performance of the fine-tuned RoBERTa model using our proposed context extraction method and other baselines. When the original article was the input context, the MRC model performed the worst, with both the lowest *F*_1_-scores and EM score on both data sets, and it also had the longest inference time. The target paragraph setting had the best *F*_1_-score and EM score on both the COVID-QA and BioASQ data sets as well as the fastest inference time on the COVID-QA data set. On the BioASQ data set, however, the exact position of the answer was not provided; therefore, we aggregated all paragraphs containing the answer as the target paragraph. As a result, the target paragraphs in the BioASQ data sets were longer than those in the paragraph retriever and context extraction methods, resulting in a longer inference time. Our proposed context extraction method for the COVID-QA data set achieved overall performance second only to the target paragraph setting. For the BioASQ data set, our method also achieved the second-best results in *F*_1_-scores and EM scores and had a slightly longer inference time than the paragraph retriever setting.

In several COVID-QA test cases, our context extraction method allowed the MRC model to predict significantly more accurately than the other baselines (Table 4). In the first example, the paragraph retriever and target paragraph methods were able to predict part of the answer, whereas the MRC model only made a somewhat relevant prediction with the original article setting. In contrast, the context extraction method allowed the MRC model to predict almost the ground truth answer (missing only the last sentence). In the second and third examples, none of the 3 baseline methods were able to predict the answer at all. However, the context extraction method enabled the MRC model to achieve high accuracy in prediction.

As mentioned in the third paragraph of the *MRC Performance With Context Extraction* subsection of the *Results* section, the BioASQ data set did not provide the exact position of the answer, and the answers were generally short (1-3 tokens) and appeared in many positions in the article. As a result, our token-matching method for identifying answer sentences not only returned answer sentences but also nonanswer sentences. Therefore, the algorithm parameters were not as optimal as the COVID-QA case, and the context extraction approach *F*_1_-score was slightly better than that of the paragraph retriever method. In terms of the EM score, our approach showed a more significant improvement compared with the original article and paragraph retriever methods. Table 5 shows 2 examples in which our method outperformed the 3 baselines by a large margin. In the first example, only the paragraph retriever method made a relatively correct prediction, whereas the other 2 baselines’ predictions were completely wrong. In the second example, all 3 baselines predicted incorrectly. Our method enabled the MRC model to predict correctly in both cases.

We further investigated the test cases where applying context extraction led to a significant change in answer prediction correctness (the difference in *F*_1_-scores was >0.5) and then counted the number of cases where context extraction led to worse or better predictions compared with the original article setting. We found that among the cases in which context extraction led to a lower *F*_1_-score, there were many cases in which the ground truth sentence as well as its surrounding sentences were selected, which means that the worse prediction was not caused by the context extraction algorithm (Table 6). Upon investigating these cases, we found two main reasons for the drop in prediction correctness:

1. Annotated ground truths are imperfect: As *F*_1_-score measures the overlap between the ground truth and prediction, when the prediction is too long or too short, it receives a low score, although it may express the same thing as the ground truth (examples 1 and 2 in Table 7).
2. Faulty behavior from the MRC model: In some cases, the MRC model was unable to predict an answer when inputting only the context (or paragraph) containing the ground truth sentence, whereas it was able to predict accurately when inputting the entire article (examples 3 and 4 in Table 7).

The second reason was interesting: reducing the context (retaining the ground truth sentence and its adjacent ones) caused the MRC model to predict significantly worse or to be unable to predict at all. We replaced the RoBERTa MRC model with the BERT for Biomedical Text Mining model [19] (BERT model fine-tuned on biomedical data) and performed the same experiments with the context extraction algorithm. Tables 8 and 9 show that the trends are consistent with those of the BERT for Biomedical Text Mining MRC model, thereby supporting our observation that the MRC model worked poorly in short and precise contexts in some cases.

**Table 4:** Examples where the proposed context extraction method significantly outperformed other methods in the Question Answering Data Set for COVID-19. The predicted answer by the machine reading comprehension model changes with different input contexts; the *F*_1_-score is calculated based on the predicted answer and the ground truth answer.

	Original article	Paragraph retriever	Target paragraph	Our approach
Question: What is the filamentous phage varion made of? Ground truth: made up of ∼2500-4000 overlapping copies of the 50-residue major coat protein, pVIII, arranged in a shingle-type lattice. Each monomer has an array of chemically addressable groups available for bioorthogonal conjugation, including 2 primary amine groups (shown in red), 3 carboxyl groups (show in blue), and 2 hydroxyl groups (show in green). The 12 N-terminal residues generally exposed to the immune system for antibody binding	Prediction: particle is enclosed by a rod-like protein capsid, ∼1000 nm long and 5 nm wide, made up almost entirely of overlapping pVIII monomers, each of which lies ∼27 angstroms from its nearest neighbor and exposes 2 amine groups as well as at least 3 carboxyl groups *F*_1_-score: 0.286	Prediction: ∼2500-4000 overlapping copies of the 50-residue major coat protein, pVIII, arranged in a shingle-type lattice *F*_1_-score: 0.356	Prediction: ∼2500-4000 overlapping copies of the 50-residue major coat protein, pVIII, arranged in a shingle-type lattice *F*_1_-score: 0.356	Prediction: ∼2500-4000 overlapping copies of the 50-residue major coat protein, pVIII, arranged in a shingle-type lattice. Each monomer has an array of chemically addressable groups available for bioorthogonal conjugation, including 2 primary amine groups (shown in red), 3 carboxyl groups (show in blue), and 2 hydroxyl groups (show in green) *F*_1_-score: 0.868
Question: Which 4 studies were included? Ground truth: phase I clinical trials on SARS or MERS^a^ vaccines	Prediction: investigation of antivirals, interferon atomization, darunavir and cobicistat, arbidol, and remdesivir use for patients with 2019-nCoV *F*_1_-score: 0.0	Prediction: antivirals, interferon atomization, Darunavir and cobicistat, arbidol, and remdesivir *F*_1_-score: 0.0	Prediction: no answer *F*_1_-score: 0.0	Prediction: phase I clinical trials on SARS or *F*_1_-score: 0.875
Question: What are examples of viral vectors for delivering vaccines? Ground truth: recombinant vaccines are based on both DNA viruses (such as fowlpox virus–based vaccines that target avian influenza virus and fowlpox virus, or vaccinia virus–based vectors against the rabies virus in wildlife) and RNA viruses (such as Newcastle disease virus–based vaccines to be used in poultry or YFV^b^-based vaccines to be used in horses against the West Nile virus)	Prediction: anthrax, hepatitis B, HIV-1, influenza, measles, SARS, malaria, and tuberculosis M. Saxena *F*_1_-score: 0.077	Prediction: Salmonella and adenovirus *F*_1_-score: 0.032	Prediction: anthrax, hepatitis B, HIV-1, influenza, measles, SARS, malaria, and tuberculosis *F*_1_-score: 0.079	Prediction: fowlpox virus–based vaccines that target avian influenza virus and fowlpox virus or vaccinia virus–based vectors against the rabies virus in wildlife and RNA viruses (such as Newcastle disease virus–based vaccines to be used in poultry or YFV-based vaccines to be used in horses against the West Nile virus) *F*_1_-score: 0.907

^a^MERS: Middle East respiratory syndrome.

^b^YFV: yellow fever virus.

**Table 5:** Examples where the proposed context extraction method significantly outperforms other methods in the Biomedical Semantic Indexing and Question Answering data set. The predicted answer by the machine reading comprehension model changes with different input contexts; *F*_1_-score is calculated based on the predicted answer and the ground truth answer.

	Original article	Paragraph retriever	Target paragraph	Our approach
Question: Which ultraconserved element is associated with embryonic stem cells’ self-renewal? Ground truth: T-UCstem1^a^	Prediction: lncRNA^b^ *F*_1_-score: 0.0	Prediction: T-UCstem1 KD^c^ *F*_1_-score: 0.667	Prediction: miR-9^d^ *F*_1_-score: 0.0	Prediction: T-UCstem1 *F*_1_-score : 1.0
Question: Which protein phosphatase has been found to interact with the heat shock protein, HSP20^e^? Ground truth: “protein phosphatase 1” and “PP1”	Prediction: PKA^f^ *F*_1_-score: 0.0	Prediction: PKA *F*_1_-score: 0.0	Prediction: PKA *F*_1_-score : 0.0	Prediction: PP1^g^ *F*_1_-score: 1.0

^a^T-UCstem1: transcribed ultraconserved stem1.

^b^lncRNA: long non-coding RNA.

^c^T-UCstem1 KD: T-UCstem1 knockdown.

^d^miR-9: microRNA-9.

^e^HSP20: heat shock protein 20.

^f^PKA: protein kinase a.

^g^PP1: protein phosphatase 1.

**Table 6:** Number of cases of significant change in predictions after context extraction in the testing data of the Question Answering Data Set for COVID-19, compared with original article baseline.

	Better (number of cases)	Worse (number of cases)	Worse because of poorly selected context (number of cases)
Paragraph retriever	25	24	2
Target paragraph	37	17	0
Our approach	33	21	4

**Table 7:** Examples of bad predictions caused by other reasons. Each row shows the model’s predictions with original article baseline and proposed context extraction method as well as the difference of input context lengths between the 2 approaches.

Number	Example
1	Question: Where was HTNV^a^ isolated from? Ground truth: from the striped field mouse *Apodemus agrarius* Baseline prediction: striped field mouse *A. agrarius* Context extraction prediction: striped field mouse *A. agrarius*, detected in part by the binding of antibodies from patient serum samples to the lung tissues of healthy, wild-caught field mice Extracted context to article length ratio (in sentences): 1:19
2	Question: What is the ultimate destination for N, for its assembly into viral particles? Ground truth: the Golgi Baseline prediction: the Golgi Context extraction prediction: the Golgi, and it traffics there via the endoplasmic reticulum-Golgi intermediate complex, also known as vesicular-tubular cluster Extracted context to article length ratio (in sentences): 1:35
3	Question: What method is useful in administering small molecules for systemic delivery to the body? Ground truth: intranasal Baseline prediction: intranasal Context extraction prediction: no answer Extracted context: intranasal entry has long been used to administer small molecules, such as proteins, for systemic delivery. Because the nasal mucosa is highly vascularized, delivery of a thin epithelium of medication across the surface area can result in rapid absorption of the medication into the blood. Therefore, siRNAs administered intranasally might be deposited in the nose, and some of them may be unable to reach the lower respiratory tract. In fact, it has been reported that intranasal application of unformulated siRNAs^b^ resulted in lower delivery efficiency and homogeneous pulmonary distribution than that achieved with intratracheal application [31]. The intranasal method is suitable for some lung diseases, such as upper respiratory infection by RSV^c^, and it also has potential for systemic delivery rather than pulmonary delivery of siRNAs. Therefore, it is important to consider the route of administration in animal studies when assessing the delivery and therapeutic efficacy of a formulation for pulmonary delivery. Careful choice of efficient delivery in response to the condition of lung diseases is necessary. Extracted context to article length ratio (in sentences): 1:45
4	Question: What past research has been done on severe, single-wave pandemics? Ground truth: after a new influenza virus (H7N9) was identified in China in 2013, a series of modeling articles described the effect of, and level of preparedness for, a severe, single-wave pandemic in the United States. Baseline prediction: a series of modeling articles described the effect of, and level of preparedness for, a severe, single-wave pandemic in the United States. Context extraction prediction: no answer Selected context: Is the world ready for a respiratory virus with high transmissibility and severity? After a new influenza virus (H7N9) was identified in China in 2013, a series of modeling articles described the effect of, and level of preparedness for, a severe, single-wave pandemic in the United States.7 In scenarios that used clinical attack rates (the proportion of individuals who become ill with or die from a disease in a population initially uninfected) of 20% to 30% (for comparison the clinical attack rate was 20% in the first year of the 2009 H1N1 pandemic), depending on severity there would be an estimated 669,000 to 4.3 million hospitalizations and an estimated 54,000 to 538,000 deaths without any interventions in the United States. The models suggested that without a vaccine, school closures would be unlikely to affect the pandemic; an estimated 35,000 to 60,000 ventilators would be needed, up to an estimated 7.3 billion surgical masks or respirators would be required; and perhaps most important, if vaccine development did not start before the virus was introduced, it was unlikely that a significant number of hospitalizations and deaths could be averted owing to the time it takes to develop, test, manufacture, and distribute a vaccine. Extracted context to article length ratio (in sentences): 1:40

^a^HTNV: Hantaan orthohantavirus.

^b^siRNA: small interfering RNA.

^c^RSV: Respiratory Syncytial Virus.

**Table 8:** Bidirectional Encoder Representations from Transformers for Biomedical Text Mining model performance with different baselines and proposed context extraction method for data of the Question Answering Data Set for COVID-19.

	*F*_1_-score	Exact match score	Time (s)
Original article	0.54	0.312	161.6
Paragraph retriever	0.591	0.344	29.3
Target paragraph	0.644	0.368	7.5
Our approach	0.597	0.346	18.7

**Table 9:** Number of cases of significant change in predictions after context extraction with Bidirectional Encoder Representations from Transformers for Biomedical Text Mining in the testing data of the Question Answering Data Set for COVID-19, compared with original article baseline.

	Better (number of cases)	Worse (number of cases)	Worse because of poorly selected context (number of cases)
Paragraph retriever	43	16	1
Target paragraph	62	8	0
Our approach	45	16	2

In summary, our proposed context extraction method helped the MRC model to predict several times faster on both the COVID-QA and BioASQ data sets. Although the *F*_1_-score did not improve significantly, we observed that our context extraction method extracted good-quality context in most cases.

## Discussion

### Principal Findings

In the result analysis, we demonstrated that our proposed context extraction method was able to extract useful context from the original lengthy article, thus slightly improving the prediction correctness of the MRC model and greatly reducing inference time. Upon further investigation, we found 2 elements that may cause the MRC model to predict poorly although the input context was well selected. Although the first reason—imperfect annotations—is hard to avoid, the second reason, which is the RoBERTa model’s strange behavior, remains uncertain.

In example 3 in Table 7, we discovered that the MRC model relied on certain phrases to find the answer. In particular, if the extra sentence “Intranasal delivery is another common method of pulmonary drug application in animal studies.” was included in the extracted context, the MRC model was able to predict “Intranasal.” We believe that the MRC model needed the phrase “Intranasal delivery” from the extra sentence to make a prediction, which was unnecessary to the human response. Similarly, in example 4, a sentence outside the extracted context was needed for the MRC model to make the prediction. In particular, when prepending one of these sentences from the original article to the extracted context, the MRC model was able to make a highly accurate prediction: “With the emergence of MERS-CoV in the Middle East, a preparedness plan was developed that included a surveillance plan, laboratory testing, and contact tracing guidance.” and “Despite the high case-fatality rate (an important measure of severity), MERS cases can be asymptomatic and mild (25% in one outbreak).” However, none of those sentences contained useful information for answering the question. We found that both the extra sentences were not relevant to the question.

In summary, this discovery presented an interesting behavior of the extractive MRC models, particularly the RoBERTa model. In some cases, the model was unable to predict the answer, although the selected context was correct; however, it predicted the answer accurately with the original lengthy article. The behavior was counterintuitive; it appeared that in these cases, the MRC model used relevant but unimportant information in the article to predict a correct answer incidentally. As a result, when the context extraction algorithm excluded such information, the MRC model was unable to find the answer. This shows that there is some difference between machine and human comprehension. It would be worthwhile to investigate this matter with other MRC models that are not a variant of BERT, such as generative MRC models. We demonstrated that our proposed context extraction method consistently extracted high-quality contexts in most cases. However, in some instances, the resulting predicted answers were poor owing to other factors.

### Conclusions

In this study, we propose a novel method for context extraction tasks in MRC in a complex domain, where multiple sentences from a long context provide the information required to answer the question accurately. We demonstrated that our proposed context extraction method works on the COVID-QA and BioASQ data sets, showing that it assists the MRC model in achieving improved prediction correctness and, more importantly, reducing the total inference time. We also observed an intriguing behavior of the MRC model: in some cases, the model performs better when presented with longer input contexts.

## Abbreviations

BERT: Bidirectional Encoder Representations from Transformers
BioASQ: Biomedical Semantic Indexing and Question Answering
COVID-QA: Question Answering Data Set for COVID-19
EM: exact match
IR: information retrieval
MRC: machine reading comprehension
QA: question answering
RoBERTa: Robustly Optimized BERT Pretraining Approach
SQUAD: Stanford Question Answering Data Set

## Data Availability

The data sets generated and analyzed during this study are available in the GitHub repository [20] and the main page of the BioASQ challenge [21].
